# Supplementary material for: Reverse transcription PCR to detect low density malaria infections
Source: Wellcome Open Res. 2022 Apr 1;6:39. Originally published 2021 Feb 22. [Version 3] doi: 10.12688/wellcomeopenres.16564.3 (PMC9086519; doi:10.12688/wellcomeopenres.16564.3)
Supplement: Peer review history

# Peer review: Reviewer response for version 3

**DOI:** 10.21956/wellcomeopenres.19701.r49634

**Published:** 2022-05-09

**Authors:** Muneaki Hashimoto

**Affiliations:** 1 Health Research Institute, National Institute of Advanced Industrial Science and Technology (AIST), Hayashi-cho, Takamatsu, 761-0395, Japan

The manuscript has been improved, then I think the paper is acceptable.

Is the work clearly and accurately presented and does it cite the current literature?

Yes

If applicable, is the statistical analysis and its interpretation appropriate?

I cannot comment. A qualified statistician is required.

Are all the source data underlying the results available to ensure full reproducibility?

Yes

Is the study design appropriate and is the work technically sound?

Partly

Are the conclusions drawn adequately supported by the results?

Partly

Are sufficient details of methods and analysis provided to allow replication by others?

Yes

Reviewer Expertise:

NA

I confirm that I have read this submission and believe that I have an appropriate level of expertise to confirm that it is of an acceptable scientific standard.

# Peer review: Reviewer response for version 3

**DOI:** 10.21956/wellcomeopenres.19701.r49635

**Published:** 2022-04-08

**Authors:** Julian Tanner

**Affiliations:** 1 School of Biomedical Sciences, Li Ka Shing Faculty of Medicine, The University of Hong Kong, Pokfulam, Hong Kong

The authors have addressed the issues originally raised.

Is the work clearly and accurately presented and does it cite the current literature?

Yes

If applicable, is the statistical analysis and its interpretation appropriate?

Yes

Are all the source data underlying the results available to ensure full reproducibility?

Yes

Is the study design appropriate and is the work technically sound?

Yes

Are the conclusions drawn adequately supported by the results?

Yes

Are sufficient details of methods and analysis provided to allow replication by others?

Yes

Reviewer Expertise:

Diagnostics

I confirm that I have read this submission and believe that I have an appropriate level of expertise to confirm that it is of an acceptable scientific standard.

# Peer review: Reviewer response for version 2

**DOI:** 10.21956/wellcomeopenres.18917.r45351

**Published:** 2021-08-06

**Authors:** Muneaki Hashimoto

**Affiliations:** 1 Health Research Institute, National Institute of Advanced Industrial Science and Technology (AIST), Hayashi-cho, Takamatsu, 761-0395, Japan

1. What I want to know is why uPCR is more sensitive than your RT-qPCR. If not, you will not be able to evaluate your RT-qPCR.
2. If contamination is the cause of ultra-sensitive detection, is there a way to prevent it? If not, your RT-qPCR in endemic areas may not be useful. Please specify where in your manuscript the reviewer's comments were or were not reflected.
3. In Abstract, you have defined the real-time reverse transcription PCR you performed as RT-qPCR. qPCR is an abbreviation for quantitative PCR. If this PCR method is qualitative, it should be clearly stated and changed to an appropriate abbreviation.

Is the work clearly and accurately presented and does it cite the current literature?

Yes

If applicable, is the statistical analysis and its interpretation appropriate?

I cannot comment. A qualified statistician is required.

Are all the source data underlying the results available to ensure full reproducibility?

Yes

Is the study design appropriate and is the work technically sound?

Partly

Are the conclusions drawn adequately supported by the results?

Partly

Are sufficient details of methods and analysis provided to allow replication by others?

Yes

Reviewer Expertise:

NA

I confirm that I have read this submission and believe that I have an appropriate level of expertise to confirm that it is of an acceptable scientific standard, however I have significant reservations, as outlined above.

# Response

**Published:** 2021-08-10

**Authors:** Peter Christensen

**Affiliations:** University of Otago, New Zealand

Thank you for your time, I hope I have addressed your questions.

1. What I want to know is why uPCR is more sensitive than your RT-qPCR. If not, you will not be able to evaluate your RT-qPCR.

RT-PCR detection of 18SrRNA is more sensitive than PCR detection of the 18SrRNA gene (Kamau et al). There is no gold standard for malaria detection other than microscopy.

 I agree that uPCR is a flawed control for sens/spec testing but the other options are worse. I could use microscopy but that has much lower sensitivity. I would need to repeat the Kamau et al. paper with changes to the sample preparation, sample volume, elution volume and kit brand if I want to determine and compare the low limits of detection.

 Although PCR is a less sensitive technology, the uPCR contains DNA from a larger sample volume and is more likely to include a parasite from patient samples with very low parasitaemias.

 To confuse matters further, both assays can detect a single parasite in a given sample volume, if the whole sample is tested. Problems arise when the PCR template is diluted and split into multiple assays.

1. If contamination is the cause of ultra-sensitive detection, is there a way to prevent it? If not, your RT-qPCR in endemic areas may not be useful. Please specify where in your manuscript the reviewer's comments were or were not reflected.

Reply: The uPCR is high throughput and used to survey many 1000s of samples regularly. The flow through contains multiple negative and positive controls (DNA extract controls and mastermix controls) per test plate. They also have sample left over to analyse and check. Using this data they found standard contamination to be a factor in some runs.

 There’s no way to determine if the unique RT-qPCR positives are true or not in this study. The RT-qPCR assay included negative controls in all runs (and they were negative). Removing the standard and making the assay qualitative is one way to remove possible contamination issues, but a positive control will always be needed and represent a source of contamination.

 I’m also not convinced that these are all contamination. RT-qPCR is a more sensitive technique, and could be detecting true positives (especially when taking an aliquot from a frozen/lysed sample – unfortunately I didn’t record which samples had more than 150ul PRBC remaining and is a limitation). There’s just no way to know when using clinical samples from asymptomatic people for evaluation. In this paper I wanted to answer a simple question “can I use a lower volume sample and a more sensitive technique to achieve similar sensitivity to uPCR?”

 I can add a point in the discussion on the importance of including multiple true negative controls, especially in field settings when using highly sensitive techniques.

1. In Abstract, you have defined the real-time reverse transcription PCR you performed as RT-qPCR. qPCR is an abbreviation for quantitative PCR. If this PCR method is qualitative, it should be clearly stated and changed to an appropriate abbreviation.

Reply: The 2 assays were run in duplicate with a high quality standard curve. There has been some discussion amongst authors as to what we are allowed to call the assay. In my opinion it is a quantitative assay (and bland-altman analysis showed reasonable agreement between the 2 assays). Unfortunately the assay doesn't follow MIQE guidelines for RT-qPCR, so one of the authors wanted it changed to RT-PCR. I recently changed all back to RT-qPCR when referring to the assay, after comments from another reviewer. I'll check and make sure it's consistent. If you have advice on this point I'm open to review.

# Response

**Published:** 2021-08-10

**Authors:** Peter Christensen

**Affiliations:** University of Otago, New Zealand

I can add this to the end of discussion is you think it's warranted.

 "It should be noted that there is no way to determine the true status of samples with positive results from a single assay type in this study. Determining whether RT-qPCR is more sensitive than uPCR in the described format is beyond the scope of this study as uPCR results were used as the gold standard for analysis."

# Peer review: Reviewer response for version 1

**DOI:** 10.21956/wellcomeopenres.18253.r44476

**Published:** 2021-07-22

**Authors:** Julian Tanner

**Affiliations:** 1 School of Biomedical Sciences, Li Ka Shing Faculty of Medicine, The University of Hong Kong, Pokfulam, Hong Kong

In this article, the authors present data on using RT-PCR on small volume clinical samples.

 This is a fairly simple, although logistically challenging, study, particularly given the present COVID situation. The study adds value to the literature around this topic and given this is:

1.  An important topic.
2.  A journal which accepts submissions Wellcome Open Research without concerning too much with impact
3.  Adds value for those looking at PCR methods in malaria-endemic regions.
4. Has suitable ethical approvals.

I would support the indexing of this article.

 Some comments to improve:

1. Figure 1 are these data in triplicate, can error bars be added?
2. Figure 2 besides Bland-Altman can further analysis be added to add some more depth to the paper?

Is the work clearly and accurately presented and does it cite the current literature?

Yes

If applicable, is the statistical analysis and its interpretation appropriate?

Yes

Are all the source data underlying the results available to ensure full reproducibility?

Yes

Is the study design appropriate and is the work technically sound?

Yes

Are the conclusions drawn adequately supported by the results?

Yes

Are sufficient details of methods and analysis provided to allow replication by others?

Yes

Reviewer Expertise:

Molecular diagnostics.

I confirm that I have read this submission and believe that I have an appropriate level of expertise to confirm that it is of an acceptable scientific standard, however I have significant reservations, as outlined above.

# Response

**Published:** 2021-07-27

**Authors:** Peter Christensen

**Affiliations:** University of Otago, New Zealand

Dear Reviewer, thanks for you time reviewing our paper.

 I have responded to your questions below.

1. Figure 1 are these data in triplicate, can error bars be added?
2. **Unfortunately not. This is one reason we called it RT-PCR not RT-qPCR (the other is a lack of internal control). We were trying to add as much concentrated sample into the reaction as possible to have detection sensitivity similar to uPCR. This was the catch, we are forced to use as much sample as possible per reaction to achieve high sensitivity, but we don't want the logistics issues of large sample volumes. So we had to decide; either we decrease the assay sensitivity to increase its quantification accuracy, or put the whole sample into the PCR reaction. As this PCR will be used for 'Hot Spot' detection at the village level, we prioritized high sensitivity over high precision.**
3. Figure 2 besides Bland-Altman can further analysis be added to add some more depth to the paper?
4. **I'm hesitant to analyse the quantifications further as they're not high quality (not triplicate, not normalised) and accurate quantification of LDMI is not particularly important (low detection limit is more important). **
5. **I'll be adding microscopy and RDT results to the next draft and more information on the species present. Some of these samples were mixed Pf+Pv infections.**

**I hope I have answered your questions fully,**

**The next draft will be out soon.**

**Best Regards,**

**Peter C**

# Peer review: Reviewer response for version 1

**DOI:** 10.21956/wellcomeopenres.18253.r44495

**Published:** 2021-06-25

**Authors:** Muneaki Hashimoto

**Affiliations:** 1 Health Research Institute, National Institute of Advanced Industrial Science and Technology (AIST), Hayashi-cho, Takamatsu, 761-0395, Japan

In this study, the authors compared RT-qPCR with uPCR to develop ultra-sensitive and quantitative Plasmodium parasite detection methods. Previously, Kamau et al. showed that the detection limit of RT-qPCR is significantly lower than that of uPCR in experiments with in vitro cultured parasites. Using RT-qPCR, the current study investigated the ability to detect the parasites with ultra-sensitivity in real-patient blood samples and examined the possibility of reducing the required blood sample volume. The authors showed that parasitemia calculated by spp. uPCR was not statistically significantly different from that calculated by Pf RT-qPCR and spp. RT-qPCR, respectively, concluding that RT-qPCR may be an alternative to uPCR. 

 My comments are below:

1. In this study, uPCR was performed as the gold standard method. The gold standard method should have sufficiently high accuracy when compared with the method undergoing evaluation. What is the rationale for uPCR being the gold standard when assessing RT-qPCR?
2. What is the cause of the false-positive results detected by RT-qPCR? For example, is there any risk of detecting rRNA of other pathogens by performing RT-qPCR? It is important to evaluate whether this method is suitable for actual blood samples
  .
3. In the paper, what should be written as RT-qPCR was written as RT-PCR. The authors need to revise this.

Is the work clearly and accurately presented and does it cite the current literature?

Yes

If applicable, is the statistical analysis and its interpretation appropriate?

I cannot comment. A qualified statistician is required.

Are all the source data underlying the results available to ensure full reproducibility?

Yes

Is the study design appropriate and is the work technically sound?

Partly

Are the conclusions drawn adequately supported by the results?

Partly

Are sufficient details of methods and analysis provided to allow replication by others?

Yes

Reviewer Expertise:

Development of malaria diagnosis devices

I confirm that I have read this submission and believe that I have an appropriate level of expertise to confirm that it is of an acceptable scientific standard, however I have significant reservations, as outlined above.

# Response

**Published:** 2021-07-27

**Authors:** Peter Christensen

**Affiliations:** University of Otago, New Zealand

Dear Reviewer,

 Thank you for your time reviewing the paper. It is very much appreciated.I have replied to each of your questions below in bold.

1. In this study, uPCR was performed as the gold standard method. The gold standard method should have sufficiently high accuracy when compared with the method undergoing evaluation. What is the rationale for uPCR being the gold standard when assessing RT-qPCR?
2. **We use uPCR because it has been fully evaluated for sensitivity and specificity in 'Imwong et al. JCM 2014'** **- 2500x more sensitive than microscopy (true gold standard) and 50x more sensitive than finger prick + PCR.** **Also, uPCR is a standard method at the SMRU and I had already produced uPCR results from these samples. My supervisor wanted to collect less sample without losing too much sensitivity, and I knew the primer set was more sensitive in RT-PCR format (Kamau et. al. JCM 2011). The samples had a lower volume cryopreserved second aliquot, so I tried the uPCR in RT-PCR format. Another reviewer has asked for the microscopy (true gold standard detection) and RDT results, which will be included in the next draft. Both are inappropriate for comparison to RT-PCR due to low sensitivity, which you'll see in the next draft.**
3. What is the cause of the false-positive results detected by RT-qPCR? For example, is there any risk of detecting rRNA of other pathogens by performing RT-qPCR? It is important to evaluate whether this method is suitable for actual blood samples
4. **The 18SrRNA primers are unique to Plasmodium, so false positives are either real positives or, more likely, contamination from the standard during plate preparation (false positive from uPCR (Imwong 2014) were found to be caused by standard contamination). Unfortunately, we were trying to include as much sample as possible to to increase sensitivity of the PCR, so we had no sample left over to investigate these false positives further.**
5. In the paper, what should be written as RT-qPCR was written as RT-PCR. The authors need to revise this.
6. **Our PCR doesn't meet the criteria for RT-qPCR so one of the authors wasn't happy naming it 'qPCR' (The PCR is not in triplicate, and doesn't have an internal control for normalising quantification). I understand the confusion, because we go on to compare quantification (which I assumed would be different but to my surprise wasn't). In my opinion the quantification of LDMI is less useful than the low limit of detection and could be removed entirely from the paper (especially without knowing the life stage - ring vs schizont will have vastly different ribosome quantities). **

I hope this answers your questions, I'll have the second draft in soon.

 Regards,

 Peter C

# Peer review: Reviewer response for version 1

**DOI:** 10.21956/wellcomeopenres.18253.r42783

**Published:** 2021-04-20

**Authors:** Gisely Melo

**Affiliations:** 1 Tropical Medicine Foundation Dr. Heitor Vieira Dourado, Instituto de Pesquisa Clínica Carlos Borborema, Manaus, Brazil

**Abstract:**

 What was the target used? Was all PCR targeted to a specific gender? What was the gold standard used? The method is not clear. Why used only *falciparum *used for the manuscript?

**Introduction:**

 What is the target? What is the comparison with methods used as the gold standard of PCR?

**Methods:**

 Why was only *Plasmodium falciparum* used? What chance does the target fail to detect other malaria? Which parasitemia of the people studied to study low parasitemia? If used, was gold pattern used as a thick blood smear or RDT? The target for RT-PCR was for *Plasmodium*? Two RT-PCRs were performed on each extract in duplicate: *Plasmodium *spp. and are they sensitive enough? Using uPCR as a gold standard would not be the most appropriate use of the thick blood smear as a control. It is not clear that he will use a method for gender and species for methodology.

**Results:**

 Using uPCR as a gold standard is not the most suitable. How to be sure of positive positives and false negatives? Why was parasitemia not compared in the studied methods to verify the comparison between the methods to verify the smallest parasitemia that it would detect? In quantification, it is more appropriate to perform correlation analysis to compare a parasitemia between the methods.

**Discussion:**

 The targets should have been described in the methodology as well. What are the limitations of the study?

Is the work clearly and accurately presented and does it cite the current literature?

Partly

If applicable, is the statistical analysis and its interpretation appropriate?

Partly

Are all the source data underlying the results available to ensure full reproducibility?

Yes

Is the study design appropriate and is the work technically sound?

No

Are the conclusions drawn adequately supported by the results?

Partly

Are sufficient details of methods and analysis provided to allow replication by others?

Yes

Reviewer Expertise:

NA

I confirm that I have read this submission and believe that I have an appropriate level of expertise to state that I do not consider it to be of an acceptable scientific standard, for reasons outlined above.

# Response

**Published:** 2021-04-22

**Authors:** Peter Christensen

**Affiliations:** University of Otago, New Zealand

Thank you for your time reviewing this paper, I appreciate the effort.

 Hopefully I can address all your comments in the next draft.

*Info on target & sensitivity specifictity data:*

 I will update the paper to include more information about the primer targets earlier and will add the previously published Sens and Spec for the Plasmodium spp. primer set used by uPCR and RT-PCR. It has been done in the original paper for both qPCR and qRT-PCR and then again for high volume uPCR. **Will adding previously published data be satisfactory?**

*Why only falciparum*:

 We used Pf only because we can produce very young rings *invitro *that are then FACS sorted for a more consistent standard *, *and because clinical vivax samples often contain late stages (which may contain more than one nucleus per infected red cell and also contain more ribosomal material). Furthermore, although there is a growing realisation of vivax malaria’s importance; the funder and clinicians at the field site still consider the sensitive and timely diagnosis of Pf a priority. Nonetheless I wish to assure the reviewer that we also would have preferred to have a Pv data set (esp. considering Pv is the most prevalent in the region); however due to the large proportion of sample per reaction (4ul of DNA template per rxn & only 20ul DNA eluted) this means we can only do two PCRs in duplicate. This is a limitation of this type of high blood volume per reaction assay. I can discuss this limitation.

*Clarify targets*:

 Only 2 new RT-PCR results were produced in this study, both target 18S RNA. One was Plasmodium genus specific, the other was *P. falciparum* specific. The original Pf classification given by uPCR used another Pf primer set from Perandin et al. JCM 2004.

 In hindsight I should have used this same Pf primer set for my RT-PCR assays. The reason I used primers from Sumari et al. is because they had been used in RT-PCR format and we originally included gametocyte data using PfS25 primers from that paper as well. We didn’t have enough sample to complete the data set, so it was removed from the paper.

*RDT and Microscopy*:

 Microscopy would be better as it is the gold standard. But the LOD is so low it would not be able to detect the majority of samples in this data set (13 of 21 are submicroscopic (<50,000 p/mL)). Unfortunately RDT data from the malaria elimination program, was found to have some important QA issues (the specific batch of PfHRP-2 RDTs provided by Global Fund)).

*Statistics:*

 I thought correlation was appropriate here as well, but our statistician sent me this paper ( https://www.ncbi.nlm.nih.gov/pmc/articles/PMC4470095/). So I used bland-altman for assessing agreement (both similarities and differences) between test measurements. Originally I wanted this assay to be yes/no and semi-quantitative and expected the analysis to **not** show agreement between tests. The reason thought this is because Kamau *et al* have already shown this primer set is less accurate at low parasitaemia when used as qRT-PCR.

*Number of LDMI*:

 I will add the number of samples that were low density (less than 50 parasites/uL) by uPCR result: 13 of 21 were LDMI.

 Again we wish to thank the reviewer for their time and expertise,

 Regards,

 Peter C
